# *Ustilago maydis* Nit2 Regulates Nitrate Utilisation During Biotrophy and Affects Amino Acid Metabolism of Galls Under Nitrogen Depletion

**DOI:** 10.1111/mpp.70148

**Published:** 2025-09-01

**Authors:** Philipp L. Lopinski, Christin Schulz, Alicia Fischer, Nadine Reichl, Timo Engelsdorf, Nadja Braun, Lars M. Voll

**Affiliations:** ^1^ Department Biology, Molecular Plant Physiology Marburg University Marburg Germany; ^2^ Microcosm Earth Center Marburg University Marburg Germany; ^3^ Center for Synthetic Microbiology (SYNMIKRO) Marburg University Marburg Germany

**Keywords:** amino acid metabolism, biotrophy, corn smut fungus, nitrate assimilation, nitrogen utilisation, nutrient acquisition

## Abstract

In previous work, we have shown that the transcription factor Nit2 plays a major role in the utilisation of non‐favoured nitrogen sources like nitrate, minor amino acids or nucleobases in saprotrophic sporidia of the basidiomycete corn smut fungus *Ustilago maydis* . Addressing the knowledge gap regarding how filamentous phytopathogens adapt to nitrogen limitation in the host plant, we employed Δ*nit2* mutants in the natural FB1 × FB2 background to identify Nit2‐regulated genes during biotrophy. We further investigated the impact of Nit2 on the physiology of leaf galls in nitrogen‐replete versus nitrogen‐limited host plants by comparative RNA‐Seq and metabolic steady state analysis. About one third of the fungal genes affected by Nit2 during biotrophy were involved in nitrogen metabolism and transport, only showing minor overlap to saprotrophic sporidia. Induction of the nitrate assimilation cluster was completely dependent on Nit2 during biotrophy. In nitrogen‐limited host plants, Δ*nit2* leaf galls accumulated nitrate and showed reduced accumulation of the nitrogen‐rich phloem transport amino acids asparagine and glutamine compared to wild‐type galls. However, total protein content in galls and pathogenicity were comparable between fungal genotypes in both nitrogen regimes. The findings of our physiological and transcriptomic analysis demonstrate that nitrate utilisation is dispensable for *U. maydis* during biotrophy and can likely be actively compensated by increased utilisation of abundant organic nitrogen sources, like asparagine, GABA and glutamine in a partially Nit2‐dependent fashion.

## Graphical abstract

The transcription factor Nit2 of the smut *Ustilago maydis* controls nitrate utilisation during biotrophy and affects steady state nitrate and amino acid contents in maize leaf galls under nitrogen depletion.

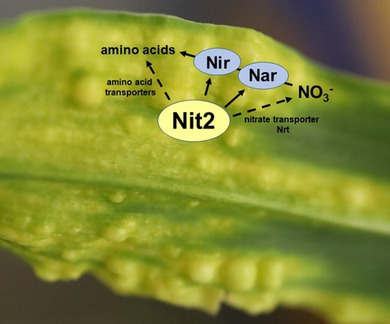

## 1 Introduction

Fungal plant pathogens fuel the development of early infection structures by mobilisation of lipid, protein and carbohydrate stores in spores (e.g., Both et al. 2005). For the ensuing establishment in the host tissue, biotrophic and hemibiotrophic pathogens have to gain access to nutrients from living host cells (as reviewed by Bezrutczyk et al. 2018). Photoassimilates like soluble sugars and amino acids have been identified as major organic nutrients for (hemi)biotrophic (fungal) leaf pathogens, but at the same time, represent important building blocks to fuel the host defence response. Thus, the competition for organic carbon and nitrogen represents a crucial battlefield in biotrophic plant–fungal interactions (as e.g., reviewed by Bolton 2009; Fernandez et al. 2014; Bezrutczyk et al. 2018).

Several reports indicate that abundant organic nitrogen sources like amino acids are predominantly tapped for the nutrition of (hemi)biotrophs. Amino acid uptake transporters have been cloned from a range of fungal pathogens, for example seminal work for the rust fungus *Uromyces fabae* (Struck et al. 2002; Struck et al. 2004) or *Cladosporium fulvum* (Solomon and Oliver 2002). Gamma‐aminobutyric acid (GABA) concentrations in the tomato apoplasm can reach up to 2 mM during *C. fulvum* infection (Solomon and Oliver 2001), and circumstantial evidence suggests that it is the major organic nitrogen source of *C. fulvum* (Solomon and Oliver 2002). Recently, the *Arabidopsis* amino acid transporter LHT1 was shown to be induced early during the host defence response against the hemibiotrophic bacterium *Pseudomonas syringae* pv. *syringae* in order to facilitate amino acid sequestration from the apoplast (Zhang et al. 2002), reflecting the aforementioned competition between host and parasite for apoplasmic amino acids. Highly interesting observations on the relevance of inorganic nitrate for nitrogen provision to phytopathogens derive from the comparative study of closely related oomycetes (Abrahamian et al. 2016; Ah‐Fong et al. 2019). In particular, the nitrate assimilation cluster of *Phytophthora infestans* was strongly induced in nitrate‐rich tomato leaves and deletion of the cluster resulted in complete loss of pathogenicity on leaves, while on nitrate‐poor potato tubers, expression of the cluster was low and pathogenicity of the cluster mutants was mildly affected (Abrahamian et al. 2016).

In the corn smut fungus *Ustilago maydis* , biotrophy is initiated during the dimorphic switch, in which two compatible haploid, saprotrophic sporidia are stimulated to mate by cues perceived from the host surface (Mendoza‐Mendoza et al. 2009; Lanver et al. 2014). Mating results in the formation of dikaryotic filamentous hyphae that develop appressoria‐like structures, which can penetrate aerial organs of the host plant maize (reviewed by Lanver et al. 2017). *U. maydis* is able to colonise meristematic tissue of maize leaves and reproductive organs with a network of inter‐ and intracellular hyphae that induce gall formation by stimulating cell divisions and hypertrophy in an organ‐specific manner (Skibbe et al. 2010; Redkar et al. 2017). Due to easier handling and shorter cultivation times of host plants, nutrient acquisition and metabolic reprogramming have been studied most intensely in leaf galls. Interestingly, infection of leaf meristems by *U. maydis* prevents photoautotrophic development of maize leaves (Doehlemann et al. 2008), including the establishment of the C_4_ syndrome (Horst et al. 2008). Consequently, infected leaves do not develop into a physiological source organ but remain a sink for organic carbon and nitrogen throughout gall development (Horst et al. 2008; Horst, Doehlemann, Wahl, Kahmann, et al. 2010). Using stable isotope labelling, Horst, Doehlemann, Wahl, Kahmann, et al. (2010) demonstrated that leaf galls outcompeted systemic sinks like young leaves for organic nitrogen and, in addition, stimulated amino acid export of systemic source leaves two‐fold. For organic carbon acquisition, the *U. maydis* high‐affinity H^+^/sucrose transporter Srt1 outcompetes host plant H^+^/sucrose transporters based on higher substrate affinity, which is pivotal for the establishment of biotrophy (Wahl et al. 2010).

Lanver et al. (2018) have elaborated a transcriptomic atlas for several stages of *U. maydis* biotrophy. Various transporters for inorganic and organic nitrogen, like the ammonia transporter *ump2* or the urea transporter *dur3‐3*, were strongly induced during biotrophy, but deleting these genes did not affect virulence (Lanver et al. 2018). In addition, more than 60% of the encoded proton coupled co‐transporters for hexoses and amino acids are induced during the biotrophy of *U. maydis* (as by re‐analysis of the data by Lanver et al. 2018), but functional data for their involvement in the provision of organic carbon and nitrogen to the pathogen are lacking to date, with one exception (Schuler et al. 2015). In contrast, the CRISPR/Cas9‐induced mutual deletion of the oligopeptide transporter genes *opt2*, *opt3* and *opt4* diminished virulence (Lanver et al. 2018), indicating that these OPTs might contribute to the uptake of organic nitrogen during biotrophy.

In saprotrophic sporidia of *U. maydis* , the utilisation of non‐favoured nitrogen sources like minor amino acids, nucleobases and nitrate was shown to be predominantly controlled by the GATA Zn finger transcription factor Nit2 (Horst et al. 2012), indicating the presence of nitrogen catabolite repression (NCR) in sporidia. The involvement of *UmNit2* homologues in NCR of heterotrophic fungal model organisms like brewer's yeast (*Saccharomyces cereviseae*), *Neurospora crassa* and *Aspergillus nidulans* is well described (reviewed in Wong et al. 2008). Several reports have demonstrated that Nit2 homologues are involved in the regulation of nitrogen utilisation in plant‐pathogenic fungi (for reviews, see Divon and Fluhr 2007; Fernandez et al. 2014). Intriguingly, most of the studied systems were hemibiotrophic ascomycetes. In *Colletotrichum lindemuthianum, Fusarium verticillioides* and *Fusarium oxysporum*, Nit2 homologues were required for full pathogenicity (Pellier et al. 2003; Divon et al. 2006; Kim and Woloshuk 2008), while the loss of Nit2 homologues in *Magnaporthe grisea* or in *C. fulvum* had little or no effect on virulence (Froeliger and Carpenter 1996; Perez‐Garcia et al. 2001), but played a role for the expression of some effector proteins. For instance, the expression of the *C. fulvum* effector protein Avr9 was found to be under the control of the Nit2 homologue Nrf1 (Perez‐Garcia et al. 2001) and five *M. grisea* pathogenicity factors were induced by nitrogen limitation in vitro (Talbot et al. 1993; van den Ackerveken et al. 1994; Donofrio et al. 2006). In the basidiomycete *U. maydis* , filamentation was delayed in Δ*nit2* mutants in the solopathogenic strain SG200, resulting in reduced pathogenicity (Horst et al. 2012). Employing strains with an arabinose‐inducible *b‐*cascade suggested that Nit2 acts downstream of the *b*‐locus in the initiation of pathogenic growth during the dimorphic switch (Horst et al. 2012), indicating the integration of nitrogen signals into the regulation of pathogenicity of *U. maydis* .

In the present report, we aim at (i) identifying Nit2‐regulated genes during biotrophy, as well as elucidating the role of Nit2 for (ii) nitrogen utilisation and (iii) pathogenicity during biotrophy of *U. maydis* as well as (iv) its possible role for the adaptation to different nitrogen regimes and nitrogen limitation in planta. Investigating the involvement of Nit2 in the regulation of the dimorphic switch and the initiation of pathogenic growth is yet out of the scope of the present manuscript.

## 2 Results

### 2.1 Establishment of a Low N Matrix for *U. maydis* Biotrophy

**FIGURE 1:**
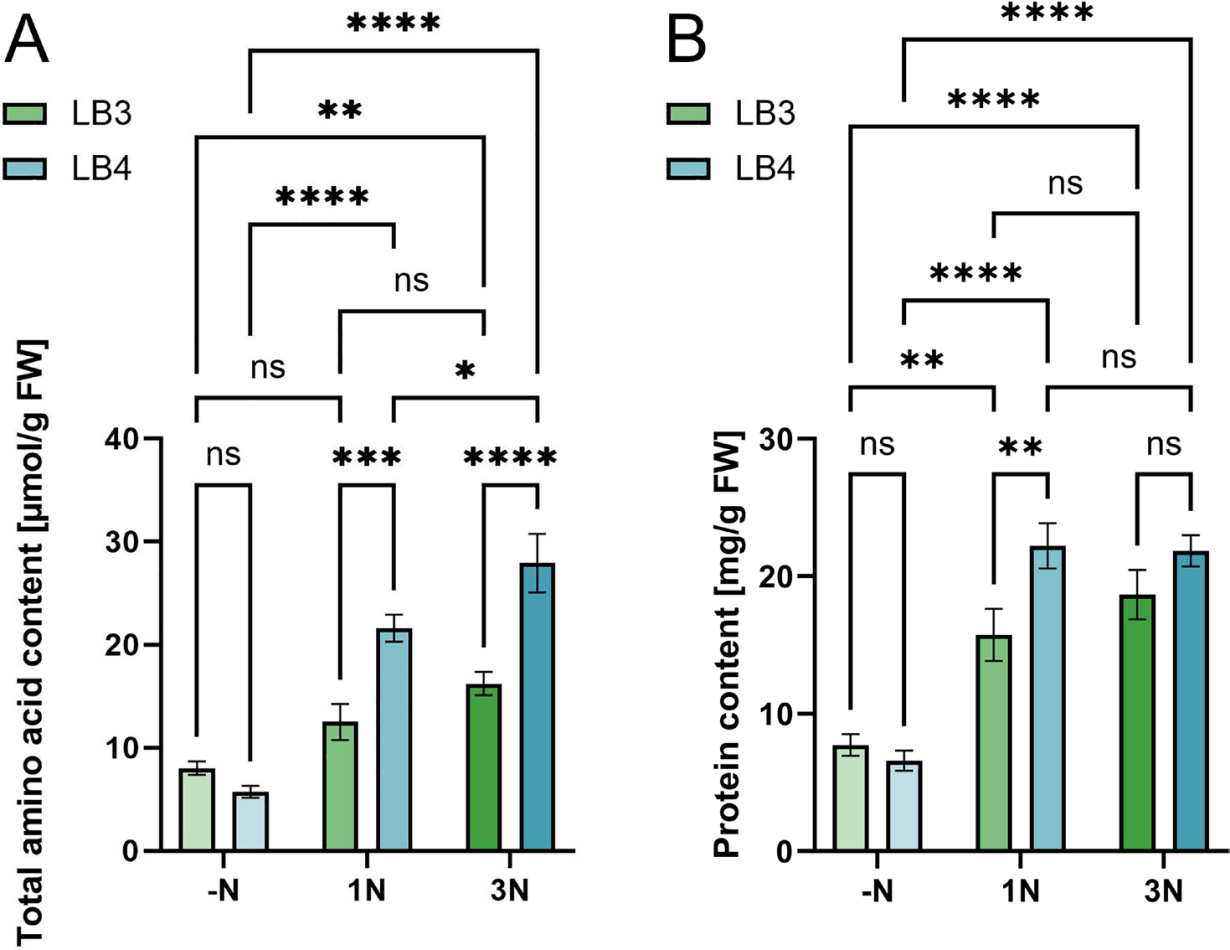
Contents of free and protein‐bound amino acids in maize leaves in the examined fertilisation regimes. The contents of (A) total free amino acids and (B) protein content in leaf 3 (LB3, green bars) and leaf 4 (LB4, blue bars) of 14‐day‐old maize plants that had been fertilised from day 7 with Hoagland nutrient solution with no nitrogen source (−N, left pairs of bars), 16 mM NO_3_^−^ and 1 mM NH_4_^+^ (1N, middle) and threefold increased inorganic nitrogen (3N, right). Data are means of four biological replicates (*n* = 4) with the error bar representing the SE. Statistical analysis was performed with a two‐way ANOVA and a Fisher LSD post hoc test (*p < 0.05; **p < 0.01; ***p < 0.001; ****p < 0.0001).

To address the role of Nit2 for the adaptation to different nitrogen regimes and, ultimately, potential nitrogen limitation in planta, we aimed at generating a high N and low N environment for *U. maydis* during biotrophy in host leaves. Therefore, we modified the fertilisation regime of the host plants and characterised the availability of free and protein‐bound amino acids as a proxy for overall availability of organic nitrogen, which we had already benchmarked in a previous study (Horst, Doehlemann, Wahl, Kahmann, et al. 2010). To this end, we fertilised maize seedlings from 7 to 14 days after sowing with 1N regular Hoagland nutrient solution containing 16 mM nitrate and 1 mM ammonium (as in Horst, Doehlemann, Wahl, Kahmann, et al. 2010, 2012), Hoagland nutrient solution with three‐fold increased inorganic nitrogen concentration (3N) and Hoagland solution without any nitrogen source (−N). In nitrogen‐replete conditions (1N and 3N), the 4th leaves showed significantly increased free amino acid contents compared to the 3rd leaves (Figure 1), which reflects that the 4th leaves are the first true C_4_ leaves. Moreover, there were no substantial differences between 1N and 3N regimes for both leaf positions, while in the −N regime, free and protein‐bound amino acid content was reduced two‐ and three‐fold compared to 1N in 3rd and 4th leaves, respectively (Figure 1). Because the main objective of our study was to investigate the impact of nitrogen limitation on gall metabolism and gene regulation in *U. maydis* Δ*nit2* mutants, we chose to further investigate galls on the 4th leaves in −N and 1N, as the effect of nitrogen availability on the biological matrix was most pronounced in this pair of conditions. Because there were comparatively minor differences in steady‐state nitrogen metabolism between 1N and 3N conditions, the 3N regime was discontinued in further experiments.

### 2.2 The Δ*nit2* Mutant Does Not Show Reduced Virulence in the Wild‐Type Background

Because the pivotal aim of our study was to elucidate Nit2‐regulated genes during late biotrophy, we decided not to use the previously described Δ*nit2* mutant in the solopathogenic SG200 background (Horst et al. 2012), as defects during late stages of biotrophy were reported for the SG200 strain (Lanver et al. 2018). We therefore generated a full‐length *nit2* knockout in the compatible FB1 and FB2 wild‐type strains, employing the Δ*nit2* deletion construct described before (Horst et al. 2012). We confirmed the deletion of the entire *nit2* open reading frame (ORF) by PCR (Figure S1) and the inability of the FB1∆*nit2* and FB2∆*nit2* mutants to utilise non‐favoured nitrogen sources (Table S1). To assess if the biological effect of *nit2* deletion on target gene regulation in FB1 and FB2 sporidia was quantitatively comparable to our previous observations for the SG200 background (Horst et al. 2012), we quantified transcript amounts of three selected Nit2‐regulated genes by reverse transcription‐quantitative PCR (RT‐qPCR) in nitrogen‐depleted conditions (−NMM) versus nitrogen‐replete conditions with ammonia as a favourite nitrogen source (AMM), using oligonucleotide primers as described previously (Horst et al. 2012). The induction of the purine transporter *UMAG_01756*, the urea permease *dur3‐3* (*UMAG_04577*) and the high‐affinity ammonium transporter *ump2* (*UMAG_05889*) on −N compared to AMM was very similar between FB2 and SG200 as well as between FB2∆*nit2* and SG200∆*nit2* (compare Table S2 to Horst et al. 2012), while induction of *UMAG_01756* and *dur3‐3* during N depletion was weaker in FB1 than in SG200 or FB2. The induction of *ump2* on −N versus AMM was comparable for all three control and all three ∆*nit2* strains, however.

**FIGURE 2:**
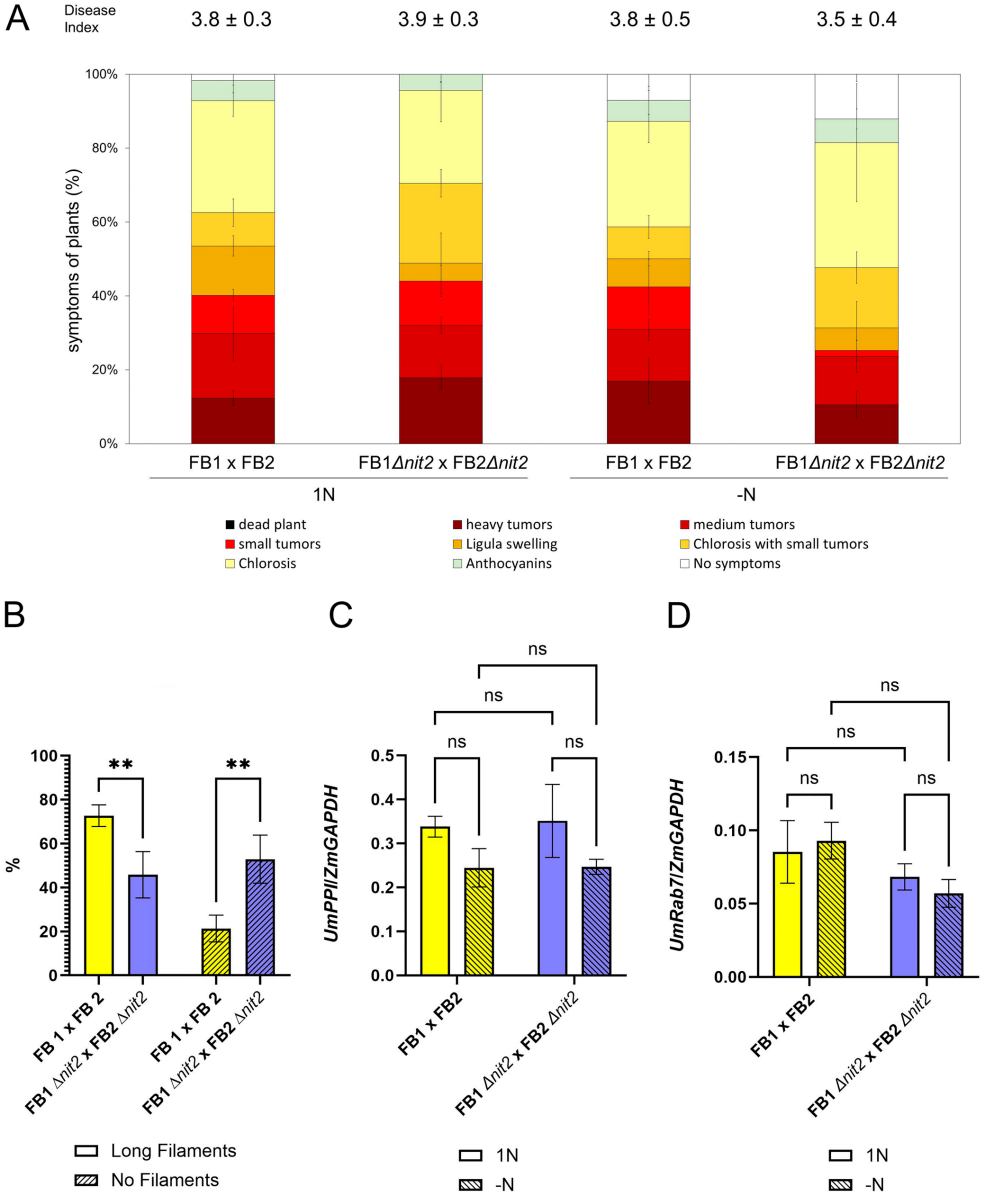
Pathogenic growth of FB1∆*nit2* × FB2∆*nit2* and FB1 x FB2 on maize leaves. (A) Disease index at 8 days post‐inoculation (dpi) with a mixture of the indicated sporidia at a titre of OD = 1. Mean values from four representative independent experiments with *N* = 15–25 plants per experimental replicate and a total number of *N* = 70–75 plants are shown ± SE. Mean values for the disease indices are given above the respective graphs ± SE. (B) Filamentation on maize leaves 18 h post‐inoculation with a mixture of the indicated sporidia at a titre of OD = 1 was assessed by calcofluor white staining of fungal structures around the injection site. *N* = 3 with a mimimum of 50 fungal structures each. The error bar represents the SE. (C) Quantitation of fungal colonisation in medium‐sized galls on the fourth leaves of 14‐day‐old plants at 8 dpi by quantitative PCR (qPCR) on genomic DNA, employing fragments of the *UmPPI* and *ZmGAPDH* gene, data are means of 6–11 (*n* = 6–11) biological replicates with the error bar representing the SE. (D) Quantification of fungal colonisation in medium‐sized galls on the fourth leaves of 14‐day‐old plants at 8 dpi by reverse transcription‐qPCR for *Ustilago maydis* and *Zea mays* housekeeping genes *UmRab7* and *ZmGAPDH*, respectively. Data are means of three to five biological replicates with the error bar representing the SE. For panels (B–D), statistical analysis was performed with a two‐way ANOVA and a Fisher LSD post hoc test (*p < 0.05; **p < 0.01; ***p < 0.001; ****p < 0.0001). Yellow bars: FB1 x FB2, blue bars: FB1∆*nit2* × FB2∆*nit2*, open bars: 1 N, hatched bars: −N.

Because we had observed delayed filamentation and reduced virulence of the SG200∆*nit2* mutant (Horst et al. 2012), we next assessed the effect of *nit2* deletion on pathogenicity in the FB1 × FB2 wild‐type background. Filamentation was also delayed 18 h after mating FB1∆*nit2* with FB2∆*nit2* compared to the wild‐type FB1 × FB2 cross (Figure 2B), but symptoms at 8 days post‐inoculation (dpi) were similar between FB1∆*nit2* × FB2∆*nit2* and FB1 × FB2 on maize plants in both N regimes (Figure 2A), which was consistent between all six replicate experiments. Because this observation was in contrast to the results we obtained in the SG200 background (Horst et al. 2012), we further investigated whether fungal proliferation in the galls was similar between FB1∆*nit2* × FB2∆*nit2* and FB1 × FB2 by two complementary assays. Quantitation of fungal genomic DNA can be regarded as a proxy for the total number of fungal nuclei in the tissue (Figure 2C), while quantification of transcripts for fungal (*UmRab7*) relative to host (*ZmGAPDH*) housekeeping genes provides an estimate for transcriptionally active fungal nuclei (Figure 2D). Both on the DNA and mRNA level, no significant differences in colonisation between mutant and wild type were detected in regular fertilised maize leaves (1N) at 8 dpi, with the mRNA data showing less variance than the genomic DNA data (Figure 2C,D, respectively).

These observations indicate that despite delayed filamentation, the ability to colonise leaf tissue remains unaltered for a cross of FB1∆*nit2* × FB2∆*nit2* versus FB1 × FB2 at a saturating infection titre (OD = 1), which is an important prerequisite for an unbiased study of Nit2‐regulated genes during biotrophy. It is highly interesting, but out of the scope of the present manuscript, to investigate the molecular basis of why pathogenicity is compromised upon loss of Nit2 in the solopathogenic SG200, but not in the wild‐type strain, while filamentation is reduced to a similar extent in both backgrounds.

Even more interesting, the fertilisation regime did not have a significant effect on disease development or host colonisation (Figure 2A,C,D), which leads to the important conclusion that Nit2 is dispensable for pathogenicity on nitrogen‐depleted host plants. However, it seems well possible that the loss of Nit2 can be compensated, making it even more interesting to investigate Nit2‐regulated genes during biotrophy.

### 2.3 Identification of Nit2‐Regulated Genes During Biotrophy

To assess the potential difference in gene regulation by Nit2 between saprotrophic sporidia and biotrophic filaments in planta, we first investigated transcript accumulation for *UMAG_01756*, *dur3‐3* (*UMAG_04577*) and *ump2* (*UMAG_05889*) representing those three Nit2‐regulated genes that we had previously validated in sporidia of the FB1∆*nit2* and FB2∆*nit2* mutant (see Table S2). In order to investigate comparable tissue in a phase of well‐established biotrophy, we harvested medium‐sized galls on the fourth leaves at 8 dpi for transcript analysis. Of these three analysed genes, only *ump2* was induced in *U. maydis‐infected* leaves of −N treated maize plants compared to leaves from 1N fertilised maize plants, indicating a partially Nit2‐dependent regulation in −N conditions in planta (Table 1). Furthermore, Nit2 seemed to be generally required for full induction of fungal *dur3‐3* in galls: while normalised *dur3‐3* transcript amounts significantly differed between FB1∆*nit2* × FB2∆*nit2* and FB1 × FB2 in 1N, a similar trend was observed in −N conditions (Table 1).

**TABLE 1:** Transcript accumulation of selected Nit2‐regulated genes in sporidia during biotrophy at 8 days post‐inoculationi by reverse transcription‐quantitative PCR.

GeneID	Annotation	1N		−N	
		FB1 × FB2	FB1∆*nit2* × FB1∆*nit2*	FB1 × FB2	FB1∆*nit2* × FB1∆*nit2*
*UMAG_01756*	Purine transporter	7.2 ± 1.0	5.3 ± 0.4	7.5 ± 0.8	6.3 ± 0.6
*UMAG_04577*	Urea permease *dur3*	0.66 ± 0.17	**0.35 ± 0.09**	0.73 ± 0.14	0.55 ± 0.08
*UMAG_05889*	*ump2*	7.2 ± 1.2	4.5 ± 1.1	**13.3 ± 2.6**	5.6 ± 0.8

*Note:* Transcript amounts are × 10^−2^ and were quantified relative to the *UmGAPDH* reference gene (Horst et al. 2012) and are means of 5–8 biological replicates ± SE. Statistical analysis was conducted with a two‐way ANOVA and a Fisher LSD post hoc test. Treatments × genotype combinations that exhibit a significant difference to the other combinations with *p* < 0.05 are shown in bold.

**FIGURE 3:**
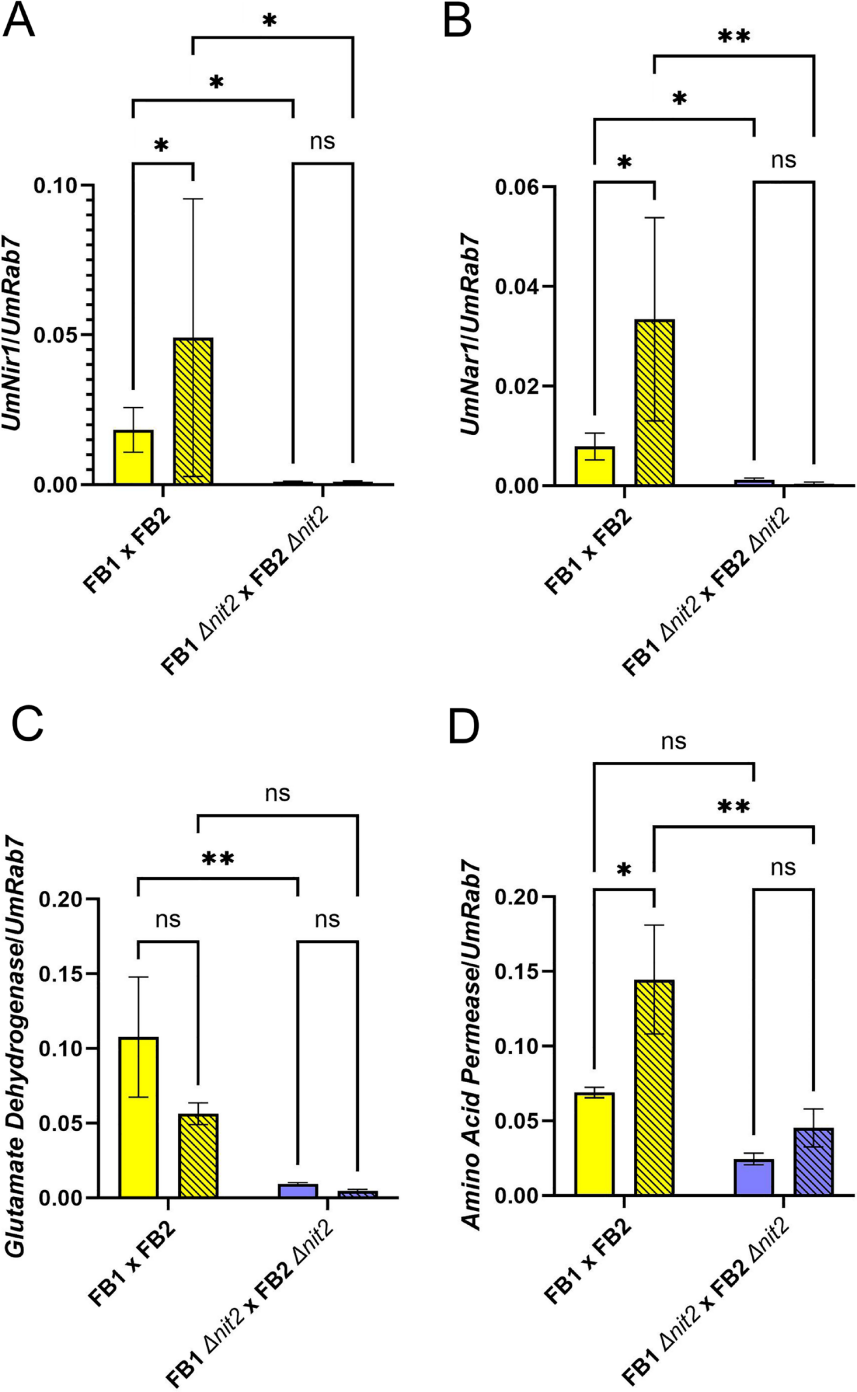
Nit2‐dependent transcript accumulation of *Ustilago maydis* genes in galls during biotrophy at 8 days post‐inoculation (dpi). Medium galls of comparable size from the fourth leaf of 14‐day‐old plants were harvested at 8 dpi and transcript amounts for the *U. maydis* genes (A) nitrite reductase *nir1* (*UMAG_11104*), (B) nitrate reductase *nar1* (*UMAG_03847*), (C) NADP glutamate dehydrogenase (*UMAG_02801*) and (D) an amino acid permease (*UMAG_00056*) were quantified in a reverse transcription‐quantitative PCR relative to the *UmRab7* reference gene. Yellow bars: FB1 × FB2, blue bars: FB1∆*nit2* × FB2∆*nit2*, open bars: 1N, hatched bars: −N. Similar results were obtained when *PPI* was used as a reference gene (Figure S2). Values are means of 3–5 biological replicates ± SE. Statistical analysis was conducted with a two‐way ANOVA and a Fisher LSD post hoc test (*p < 0.05; **p < 0.01).

Taken together, we did not observe a strong Nit2‐dependent regulation for all three tested genes in low N during biotrophy, suggesting a different set of Nit2 target genes in planta. Therefore, we performed RNA‐Seq of *U. maydis* genes in medium‐sized leaf galls at 8 dpi on plants raised in 1N and −N conditions to screen for Nit2‐regulated genes at a stage of well‐established biotrophy (Table S3). In 1N conditions, we found 34 *U. maydis* genes showing a more than 2‐fold reduction in transcript amount in FB1∆*nit2* × FB2∆*nit2* compared to FB1 × FB2 galls (Table S3), with 11 of these 34 genes, that is, 32%, being involved in organic nitrogen metabolism, as based on annotation. In −N conditions, 44 *U. maydis* genes showed a more than 2‐fold reduction in FB1∆*nit2* × FB2∆*nit2* compared to FB1 × FB2 galls (Table S3). Thirteen of these genes, approx. 30%, were associated with nitrogen metabolism. Among all genes, transcripts of *nit2* showed the strongest reduction of approx. 1000‐fold in all 1N and −N samples from FB1∆*nit2* × FB2∆*nit2* galls compared to FB1 × FB2 ∆*nit2* galls, confirming a functional *nit2* knockout during biotrophy at 8 dpi. Notably, nitrite reductase *nir1* (*UMAG_11104*) transcripts were more than 30‐fold diminished in ∆*nit2* galls in both N regimes, while nitrate reductase *nar1* (*UMAG_03847*) transcripts were almost 10‐fold reduced in ∆*nit2* galls compared to FB1 × FB2 wild‐type galls in N‐depleted conditions (Table S3). In addition, the nitrate transporter *nrt* (*UMAG_11105*) showed an almost 30‐fold decrease in ∆*nit2* galls compared to wild‐type galls in −N, and an 8‐fold decrease in 1N conditions (Table S3). Therefore, we took a closer look at *nar1*, *nir1* and two other genes by RT‐qPCR that showed the strongest Nit2‐dependent regulation in both N regimes in an independent experiment (Figure 3).

In galls at 8 dpi, the *U. maydis* *nir1* and *nar1* genes were induced in the wild‐type FB1 × FB2 strain under host N depletion (Figure 3A,B), confirming the nitrogen‐dependent regulation of *nir1* and *nar1* during biotrophy of *U. maydis* . Furthermore, the results of the RT‐qPCR confirmed the strong reduction in *nir1* and *nar1* transcript amounts in ∆*nit2* galls, especially in −N conditions (Figure 3A,B). Similar results for *nir1* and *nar1* were obtained when data were normalised to *PPI* instead of *Rab7* transcript amounts (Figure S2). Compared to the wild type, ∆*nit2* mutant galls showed an almost 20‐fold reduction in *nir1* transcript and a 6‐fold reduction in *nar1* transcript amount in regular fertilised plants (1N), while a more than 50‐fold reduction and even a 93‐fold reduction in *nir1* and *nar1* transcripts were evident in −N, respectively (Figure 3A,B), with the *nir1* transcript amounts being close to the detection limit in samples of ∆*nit2* galls. An N‐dependent regulation was absent for *nir1* and *nar1* in ∆*nit2* during biotrophy, indicating that nitrate utilisation might generally be hampered in galls caused by ∆*nit2* mutants.

Transcript abundance for *U. maydis* NADP glutamate dehydrogenase (*UMAG_02801*) and the amino acid transporter *UMAG_00056* were also found to be diminished in ∆*nit2* compared to wild‐type galls at 8 dpi by RT‐qPCR (Figure 3C,D). NADP glutamate dehydrogenase transcripts were about 10‐fold reduced in ∆*nit2* galls in both N regimes (Figure 3C), while *UMAG_00056* transcripts accumulated 3‐fold less in ∆*nit2* galls (Figure 3D). The amino acid permease *UMAG_00056* still showed more than a 2‐fold induction in ∆*nit2* on −N compared to 1N‐fertilised host leaves, indicating the presence of other transcriptional regulators than *nit2*. In contrast, NADP glutamate dehydrogenase *UMAG_02801* rather appeared to be slightly transcriptionally repressed in N‐depleted host leaves compared to 1N‐fertilised leaves. While it can be assumed that the loss of Nit2 has substantial effects on nitrate utilisation of *U. maydis* during biotrophy, the effect on the utilisation of minor amino acids seems less apparent. Nevertheless, GO term analysis revealed that the categories ‘transport’ and ‘transmembrane transport’ were enriched among the *U. maydis* transcripts downregulated in ∆*nit2* compared to wild‐type galls in both N regimes (Figure S3). We therefore analysed relative transcript abundance of all annotated amino acid transporters in the RNA‐Seq datasets for wild type and ∆*nit2* in 1N and −N‐grown hosts (Figure S4). In the *U. maydis* wild type at 8 dpi, transcripts for *UMAG_00056* were the fourth and the fifth most abundant in 1N and −N, respectively, representing about 5% of all transcripts for amino acid transporters. For both wild type and ∆*nit2*, the top five candidates represented about half of all transcripts for amino acid transporter genes, with *UMAG_02549* and *UMAG_06012* alone amounting to 25% of the total. In −N, however, this pattern remained largely the same for ∆*nit2*, while for the wild type the share of *UMAG_02549* and *UMAG_06012* increased to almost 50% (Figure S4), suggesting potential differences in amino acid uptake and metabolism in ∆*nit2* and wild‐type galls.

### 2.4 Analysis of Steady State Amino Acid Contents in Wild‐Type and ∆*nit2 Galls*

**FIGURE 4:**
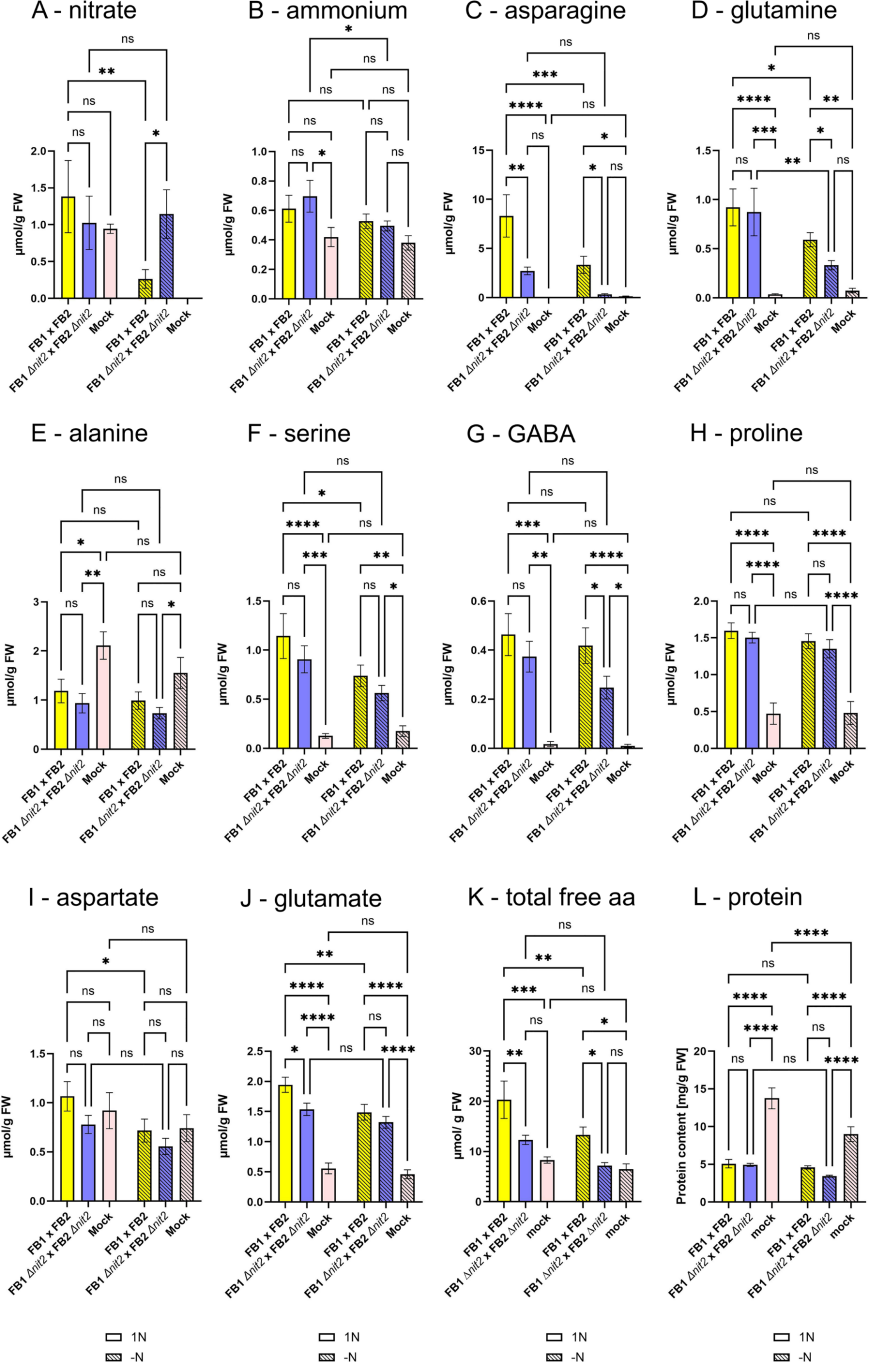
Steady‐state contents of major inorganic nitrogen sources as well as free and bound amino acids in medium‐sized galls at 8 days post‐inoculation (dpi). (A) Nitrate, (B) ammonium, (C) asparagine, (D) glutamine, (E) alanine, (F) serine, (G) γ‐aminobutyric acid (GABA), (H) proline, (I) aspartate, (J) glutamate, (K) total free amino acid and (L) protein content. Medium galls of comparable size from the fourth leaf of 14‐day‐old plants were harvested at 8 dpi. Yellow bars: FB1 × FB2, blue bars: FB1∆*nit2* × FB2∆*nit2*, pink bars: non‐infected control leaves, open bars: 1N, hatched bars: −N. Values are means of 6–7 biological replicates ± SE for 1N, 8–11 biological replicates ± SE for −N and 4–6 biological replicates for healthy control leaves. Data from one representative out of three independent experiments are shown. Statistical analysis was conducted with a two‐way ANOVA and a Fisher LSD post hoc test (*p < 0.05; **p < 0.01; ***p < 0.001; ****p < 0.0001).

Steady‐state contents of free Asn, Gln and Glu (involved in N assimilation), GABA and Pro (derived from Glu) and the photorespiratory intermediate Ser were substantially increased in galls formed by both fungal genotypes on nitrogen‐replete maize plants at 8 dpi (Figure 4C–J), while contents of the C_4_ marker amino acid Ala were reduced by 50% in galls compared to mock control leaves on 1N‐fertilised plants (Figure 4E), similar to what was previously described in Horst, Doehlemann, Wahl, Kahmann, et al. (2010) for galls formed by SG200. In 1N conditions, only Asn contents were diminished in ∆*nit2* compared to wild‐type galls, while the steady state contents of all other amino acids did not differ between galls caused by the two genotypes in 1N.

In –N conditions, the contents of the major free amino acids Asn, Gln, Glu, GABA, Pro and Ser as well as total free amino acid content were increased in wild‐type galls compared to mock control leaves in −N, albeit to a lesser extent than in 1N (Figure 4), indicating that a strong flux of organic nitrogen is maintained into galls in N‐depleted host plants. While the contents of most free amino acids were similar between wild‐type and ∆*nit2* galls, the contents of Asn, Gln and GABA were decreased in ∆*nit2* compared to wild type galls in −N conditions, suggesting altered amino acid metabolism in ∆*nit2* galls. In turn, nitrate accumulated fourfold in ∆*nit2* compared to wild‐type galls in nitrogen‐depleted conditions. Furthermore, nitrate was not detectable in mock control leaves in −N (Figure 4A), indicating that nitrate is allocated to galls in −N, but cannot be utilised efficiently in ∆*nit2* galls. For comparison, nitrate contents were similar in ∆*nit2* galls, wild‐type galls and mock control leaves of nitrogen‐replete plants (Figure 4A). Free ammonium was slightly increased in galls compared to mock control leaves in both N regimes, with no difference between fungal genotypes (Figure 4B).

The observed free amino acid contents point towards confined differences in organic nitrogen metabolism between ∆*nit2* and wild type galls. To examine if this has an impact on the capacity for protein synthesis, that is, the pool size of protein bound amino acids, protein contents were determined (Figure 4L). Total leaf protein content was 25% reduced in −N compared to 1N‐grown mock‐treated fourth leaves, likely being a result of reduced nitrogen availability. Total leaf protein content was further reduced in galls, but did not differ between the N regimes and fungal genotypes (Figure 4L), indicating no substantial limitation in the availability of free amino acids for protein biosynthesis in galls of FB1∆*nit2* × FB2∆*nit2*.

## 3 Discussion

### 3.1 Disentangling the Role of Nit2 in the Control of Pathogenicity and Nitrate Utilisation During Biotrophy

For the utilisation of nitrate, a cluster comprising three genes, the nitrate transporter *nrt*, nitrate reductase *nar1* and nitrite reductase *nir1*, is required in *U. maydis* (McCann and Snetselaar 2008; Khanal et al. 2021). In sporidia, all three genes of the cluster, and hence nitrate utilisation, were found to be under the predominant control of Nit2 in conditions when NCR was released, that is, on nitrate minimal medium or upon nitrogen depletion (Horst et al. 2012). Studies on the role of nitrate utilisation for *U. maydis* biotrophy have not only been hampered by the fact that ∆*nit2* mutants exhibit delayed and reduced filamentation in the solopathogenic SG200 background (Horst et al. 2012), but also because defects in any of the three structural genes of the nitrate utilisation cluster result in nonpathogenic strains or strains with strongly reduced virulence (e.g., Khanal et al. 2021, own unpublished results). In other filamentous phytopathogens, defects in nitrate utilisation did not affect pathogenicity in hemibiotrophic ascomycete fungi like *C. fulvum* , *M. grisea* and *Stagonopora nodorum* (Cutler et al. 1998; Lau and Hamer 1996; Talbot 1990). Deleting the nitrate assimilation cluster in the oomycete *P. infestans* completely abrogated virulence in nitrate‐rich tomato leaves, while virulence on nitrate‐poor potato tubers was merely affected (Abrahamian et al. 2016). In most of the studied systems, nitrate utilisation was dispensable for full virulence.

In the FB1 × FB2 background reported here, disruption of *nit2* also led to a strong reduction in filamentation as in the solopathogenic strain SG200, but did not reduce pathogenicity in the further course of the interaction. Given the high infection titre of OD = 1, it is not surprising that a quantitative, minor delay in filamentation does not result in diminished colonisation, because the number of timely successful penetration attempts will still be sufficient to allow for efficient colonisation in the absence of further defects. This observation further suggests two things. First, this indicates that the artificial regulation of *b* in the solopathogenic SG200 strain might exert side effects on either penetration or post‐penetration growth. Interestingly, nitrogen limitation in haploid sporidia led to ectopic activation of bE and bW in the 1/2 (*a1b1*, as FB1) but not in the 2/9 (*a2b2*, as FB2) background (Khanal et al. 2021), indicating different recruitment of Nit2 during the induction of filamentation by the two *b* alleles. Second, the observed loss of pathogenicity for mutants in nitrate metabolism might probably solely be due to a compromised early infection phase (as reported by Khanal et al. 2021). It will be highly interesting to resolve how the interaction of Nit2 and *b* proceeds on the molecular level, but this is clearly out of the scope of the present manuscript, which is meant to describe the role of Nit2 for the regulation of nitrogen utilisation during biotrophy.

To this end, we have observed that *nar1* and *nir1* are controlled by Nit2 during the biotrophic phase of *U. maydis* , both in regularly fertilised host leaves (1N) and in nitrogen depleted host leaves (−N), with consistent data obtained in independent experiments by RNA‐Seq and RT‐qPCR. Both *nar1* and *nir1* transcripts were close to or at the detection limit in ∆*nit2* galls, indicating that nitrate utilisation during biotrophy depends on Nit2. The degree of control exerted by Nit2 on *nar1* and *nir1* during biotrophy is much stronger in comparison to sporidia (Horst et al. 2012). It appears tempting to speculate that such a strong transcriptional control of nitrate utilisation by Nit2 might have implications for the adaptation to nitrogen availability, but Nit2 was only 1.6‐fold induced in wild type galls in −N compared to 1N conditions on the transcriptional level (Table S3), suggesting that either post‐translational regulation of Nit2 prevails, or that the compared N regimes do not lead to a strong difference in the regulation of Nit2. In order to test if *nit2* induction can be further increased during biotrophy, nitrogen limitation in the host plant could be exacerbated by applying −N conditions to plants cultivated on sandy or peaty substrates or by low light.

### 3.2 Nitrate Utilisation Is Dispensable for Pathogenicity During Biotrophy

In order to unlock viable approaches for disease protection, crucial processes in the nutritional strategies of individual pathogens need to be identified in planta. Biotrophic phytopathogens rely on the active provision of nutrients, organic carbon and nitrogen sources by the host tissue, for which they actively reprogramme host metabolism (for reviews see Fernandez et al. 2014; Bezrutczyk et al. 2018). Restricting supply of essential carbon building blocks from the host can mean a valuable addition to broad‐spectrum and durable resistance, as exemplified by the wheat *Lr67* gene (Moore et al. 2015) and the rice *xa13* gene (Chu et al. 2006; Yang et al. 2006; for a compilation see e.g., Bezrutczyk et al. 2018).

In the vast majority of analysed pathosystems, fungal (hemi)biotrophs divert abundant organic nitrogen sources like amino acids as their preferred nitrogen source (see introduction), while the role of nitrate for pathogen nutrition is less well investigated. According to Solomon and Oliver (2001), the tomato apoplasm contains 4–5 mM nitrate, which makes it much more abundant than any proteinogenic amino acid, which ranges between 0.1 and 0.7 mM. GABA concentrations in the tomato apoplasm can reach up to 2 mM during *C. fulvum* infection (Solomon and Oliver 2001), and besides GABA being a potent reactive oxygen species (ROS) quencher, circumstantial evidence suggests that it is the major organic nitrogen source of *C. fulvum* (Solomon and Oliver 2002). However, the contribution of nitrate to *C. fulvum* nitrogen acquisition has not been investigated. In the oomycete *P. infestans* , the nitrate assimilation cluster was strongly induced in nitrate‐rich tomato leaves but not in nitrate‐poor tubers (Abrahamian et al. 2016). Consequently, the deletion of the nitrate assimilation cluster resulted in a complete loss of pathogenicity on leaves, while pathogenicity was only mildly affected in tubers (Abrahamian et al. 2016). A subsequent study indicated that transcript accumulation of nitrate reductase and a homologue of the Nit2 repressor from filamentous fungi, NMR, were inversely correlated (Ah‐Fong et al. 2019), indicating the control of nitrate utilisation during the pathogenic growth of *P. infestans* by NCR. In addition, metabolomic analysis of infected potato tubers indicated that several pathways for minor amino acid biosynthesis were strongly induced during the biotrophic phase of *P. infestans* (Ah‐Fong et al. 2019), while 20% of all amino acid transporters were strongly expressed in tubers (Abrahamian et al. 2016), indicating that *P. infestans* might preferentially utilise free amino acids as a nitrogen source in tuber tissue. Taken together, this indicates that *P. infestans* seems capable of adapting itself to utilise nitrate as a predominant N source in response to the biological matrix.

In the maize apoplasm, nitrate and total amino acids concentrations were found to be similar at around 1.3 mM, while the leaf content of free amino acids was determined to be five times higher, that is, 9.8 μmol/g FW, than the leaf nitrate content of 1.8 μmol/g FW (Lohaus et al. 2000). In the present study, we observed a free amino acid content of 8.3 μmol/g FW and a leaf nitrate content of around 1.0 μmol/g FW in mock control leaves in the 1N regime, which is in good accordance with the data by Lohaus et al. (2000). Therefore, we assume that the concentrations of amino acids and nitrate in the leaf apoplasm can also be deemed comparable between these two studies. While nitrate content was similar in galls and mock leaves in 1N conditions, nitrate could not be detected in mock leaves of nitrogen‐depleted plants, indicating a strong effect of the −N regime on foliar nitrate. Interestingly, nitrate was still detectable in galls under −N conditions, albeit nitrate contents in wild‐type galls were seven‐fold reduced compared to 1N. In contrast, the nitrate content of ∆*nit2* galls remained similar in both N regimes. This suggests that (i) *U. maydis* colonisation in galls generates a sink for nitrate and that (ii) this nitrate cannot be utilised by ∆*nit2* galls. Combined transcriptome and NR activity analysis by our group clearly indicated that nitrate assimilation by host cells is strongly reduced in galls compared to mock control leaves at 8 dpi (Horst, Doehlemann, Wahl, Kahmann, et al. 2010), which is probably caused by an overall suppression of photosynthetic development (Doehlemann et al. 2008) and associated pathways for organic carbon and nitrogen assimilation (Horst et al. 2008; Horst, Doehlemann, Wahl, Kahmann, et al. 2010). A parallel RNA‐Seq analysis of maize genes did not show differential regulation of the host nitrate reductase gene *Nar1S* between ∆*nit2* galls and wild‐type galls in 1N or −N (not shown). Because the ∆*nit2* strain does not contribute any NR activity, we can conclude that diminished nitrate assimilation in host leaves causes nitrate accumulation in ∆*nit2* galls in −N conditions. As we observed five‐fold less nitrate accumulation in galls caused by the wild type compared to ∆*nit2* galls, we conclude that the *U. maydis* wild type assimilates nitrate during biotrophy. And because fungal colonisation did not differ between the two strains in −N, nitrate assimilation by *U. maydis* must be dispensable for virulence and biotrophy in nitrogen‐depleted host plants.

### 3.3 Nit2 and Its Downstream Genes Are Dispensable for Pathogenicity During Biotrophy

While we found that nitrate assimilation by *nar1* and *nir1* was entirely under the control of Nit2 during biotrophy, we also observed that more than 30% of all differentially regulated genes between ∆*nit2* and wild‐type galls were associated with organic nitrogen metabolism (see Table S3). Most of these genes differed between 2‐ and 8‐fold between the fungal genotypes, indicating that they were only partially controlled by Nit2 during biotrophy. Transcripts for three amino acid transporter genes, the genes for the GABA permease UGA4 related transporter *UMAG_03522*, and the two general amino acid transporters *UMAG_00056* and *UMAG_00343* were less abundant in ∆*nit2* galls compared to wild‐type galls in −N conditions. In contrast, only *UMAG_00056* and the general amino acid permease *UMAG_06012* were less expressed in ∆*nit2* galls in 1N conditions, indicating that Nit2 might play a role in remodelling amino acid uptake specificity of biotrophic hyphae in response to N availability. In fact, these adjustments in the transportome might prevent an effect on pathogenicity, as host colonisation was found to be comparable between FB1 × FB2 and FB1∆*nit2* × FB2∆*nit2* as well as between nitrogen regimes at 8 dpi. Therefore, one or a group of these differentially regulated amino acid transporters might have an effect on pathogenicity when knocked out.

Other organic nitrogen sources might also contribute to nitrogen provision to *U. maydis* . The three putative oligopeptide transporters *opt2*, *opt3* and *opt4* were strongly induced during biotrophy and simultaneous CRISPR/Cas9‐mediated knockout of all three homologues specifically affected pathogenicity in the late biotrophic phase (Lanver et al. 2018). OPTs can have a broad but distinct substrate specificity, ranging from dipeptides to glutathione, phytochelatins and oligopeptides (e.g., Osawa et al. 2006; Ito et al. 2013). Pending the functional characterisation of these three transporters in vivo, we cannot rule out that OPTs have additional vital functions than providing organic nitrogen during *U. maydis* biotrophy.

The same study by Lanver et al. (2018) revealed that, in contrast, knockout of all three *dur3* urea transporter homologues did not affect compatibility in nitrogen‐replete host plants. While two of the three encoded *dur3* urea permeases, *dur3‐2* and *dur3‐3*, were even partially controlled by Nit2 in 1N conditions, they were not found to be differentially regulated between the strains in galls in the −N regime. However, we investigated if *dur3* might affect compatibility in −N conditions, but the pathogenicity of the *dur3*‐triple knockout strain was similar to wild type in both nitrogen regimes (Figure S5).

### 3.4 Nit2 Affects Amino Acid Metabolism of Galls During Biotrophy

Although the impact of Nit2 deletion on pathogenicity was largely absent in both tested N regimes, effects on steady‐state levels of nitrate (see above) and some major amino acids in galls were evident, especially in the −N regime. First of all, this observation shows that Nit2‐dependent changes in fungal metabolism affect overall gall metabolism, and secondly, this indicates that gall metabolism is affected more strongly by Nit2‐regulated genes in nitrogen‐depleted compared to nitrogen‐replete host plants. Significant differences between ∆*nit2* galls and wild‐type galls were observed for the nitrogen‐rich amino acids Asn and Gln, as well as for GABA. Asn and Gln are known to accumulate strongly in galls (Horst, Doehlemann, Wahl, Kahmann, et al. 2010) and represent major amino acids for source–sink transport in the phloem sap of maize (Wiener et al. 1991). We have demonstrated before that leaf galls represent strong sinks for organic nitrogen exported from lower systemic leaves (Horst, Doehlemann, Wahl, Kahmann, et al. 2010), so we can assume that Asn and Gln are unloaded together with phloem‐resident nitrate into the galls, where they are preferentially taken up by fungal hyphae. This idea is corroborated by the observation that after feeding of ^1^H‐Asn, metabolisation into organic acids and phosphorylated intermediates occurred much faster in galls compared to healthy control leaves (Horst, Doehlemann, Wahl, Hofmann, et al. 2010). Such assumptions should be interpreted with care, because we cannot discriminate the contribution of host and pathogen to the total free amino acid pool in galls. Because fungal colonisation and gall size were comparable between the sampled ∆*nit2* and wild‐type galls, we can assume that the contribution of host metabolism is similar in both situations. Therefore, the specific reduction in steady‐state contents of Gln and especially Asn in ∆*nit2* galls compared to wild‐type galls in −N conditions strongly suggests that this reflects increased uptake of Asn by ∆*nit2* hyphae, probably as a compensation for the deficiency in nitrate uptake. Gln might even be converted to GABA in the apoplast and taken up by fungal hyphae as GABA, similar to the situation in *C. fulvum* discussed above (Solomon and Oliver 2001; Solomon and Oliver 2002). In support of this idea, the *U. maydis* homologue of the yeast GABA permease UGA4 (*UMAG_03522*) is one of the amino acid transport genes that show clear Nit2‐dependent induction in –N during biotrophy, and *UMAG_03522* was also induced 1.8‐fold in wild‐type hyphae in response to nitrogen depletion (Table S3). If we assume that GABA uptake is partially mediated by Nit2 in –N conditions, it cannot be explained; however, why GABA accumulation in ∆*nit2* galls is reduced compared to wild‐type galls under these conditions.

The final question is whether we have obtained any indication that ∆*nit2* is nitrogen limited in −N conditions. First of all, and most importantly, host colonisation did not differ between the fungal genotypes in nitrogen‐depleted conditions, which speaks against nutrient limitation of the ∆*nit2* strain. In addition, steady‐state analysis failed to reveal differences in protein content between ∆*nit2* and wild‐type galls. Hence, it seems unlikely that the availability of amino acid building blocks is more limited in ∆*nit2* than in the wild type.

But how can we explain the more than two‐fold decrease in total protein content in gall tissue compared to mock leaves? In general, leaf protein is dominated by the photosynthetic apparatus. In galls, however, the expression of photosynthetic genes is strongly repressed at 8 dpi (Doehlemann et al. 2008), and hence the accumulation of proteins involved in the photosynthetic machinery and the Calvin‐Benson cycle will be strongly reduced. Likewise, the strong decrease in total protein content in mock leaves under nitrogen limitation compared to 1N conditions is likely to reflect reduced availability of amino acid building blocks for the synthesis of the photosynthetic machinery and enzymes of major carbohydrate metabolism.

The role of potential nitrogen limitation for pathogenicity of fungal phytopathogens, especially for biotrophs, has long been debated in the last decades. Although this work does not provide final answers about whether nitrogen limitation occurs during the initial post‐penetration phase and if this might affect virulence, we report several seminal findings in that

1.
*Ustilago maydis* is unlikely to suffer from nitrogen limitation in the host under natural conditions
2. The utilization of nitrate is dispensable for *U. maydis* during biotrophy
3. The GATA transcription factor Nit2 regulates a different, but partially overlapping set of genes during biotrophy compared to saprotrophic sporidia
4. Nit2 and its effect on gene regulation during biotrophy is dispensable for full virulence
5. The utilisation of nitrate and amino acids during biotrophy might be regulated based on availability

It remains a challenge for the future to determine the synergistic and antagonistic effects of N limitation and *U. maydis* galls on the metabolic reprogramming of maize leaves in order to increase our knowledge on how the fungal biotrophs subvert host metabolism. It seems especially rewarding to investigate how the flux of amino acids from host source leaves to strong N sinks like galls is affected in low N regimes. Likewise, resolving the contribution of (individual) amino acid, oligopeptide and nucleotide transporters to organic N supply to the fungus will improve our mechanistic understanding of the underlying metabolic reprogramming.

## 4 Experimental Procedures

### 4.1 Plant and Fungal Material and Growth Conditions

*Ustilago maydis* strains FB1∆*nit2* and FB2∆*nit2* were generated from FB1 and FB2 isolates (Banuett and Herskowitz 1994) by deleting the entire *nit2* ORF with the construct described in Horst et al. (2012). The *dur3‐1*,*2*,*3* triple knockout strain was generously provided by Regine Kahmann (MPI for Terrestrial Microbiology, Marburg). Fungal strains were maintained on YEPS_light_ liquid medium and plates, while sporidia for plant infections were collected from potato dextrose plates (Tsukuda et al. 1988).

Maize cv. Early Golden Bantam was germinated in moist boxes at 28°C for 2 days, before seedlings of comparable size were transferred to P‐type soil (Fruhstorfer Erde) and plants were grown in phytochambers at a 14 h light (28°C, RH 50%–60%) and 10 h dark (20°C, RH 60%–70%) regime at a PFD of 350 μmol·m^−2^ s^−1^. We fertilised maize seedlings from 7 to 14 days after sowing with 1N regular Hoagland nutrient solution containing 16 mM nitrate and 1 mM ammonium (Table S4), or Hoagland nutrient solution with three‐fold increased inorganic nitrogen concentration (3N) or Hoagland nutrient solution without any nitrogen source (−N), as indicated. Plant infection was conducted 7 days after transfer to soil as described (Gillissen et al. 1992; Horst, Doehlemann, Wahl, Kahmann, et al. 2010). To this end, equal volumes of compatible fungal sporidia (OD = 1 each) were mixed and syringe‐injected into the stems approx. 2 cm above ground, which resulted in the local infection of leaf 4 and sometimes also leaf 5. The same amount of water was injected into mock control plants. Disease indices were essentially calculated as described by Schilling et al. (2014), but employing disease categories ranging from 0 (no symptoms) to 8 (dead plant), as indicated in the respective figures.

### 4.2 Isolation of Total RNA and Transcript Analysis by RNA‐Seq and RT‐qPCR

Total RNA of gall material harvested at 8 dpi was extracted with the method of Chomczynski and Sacchi (2006) and RNA‐Seq analysis was performed by Novogene (Cambridge, UK), while RT‐qPCR validation of *U. maydis* genes was performed as described by Horst et al. (2012), employing the primers described therein. Prior to their use in RT‐qPCRs with samples from infected leaf material, all primer pairs were validated not to produce any products from non‐infected leaf samples. During sample analysis by RT‐qPCR, negative control samples from mock‐treated control leaves were always analysed in parallel to rule out the contribution of plant derived amplificates.

### 4.3 Isolation of Genomic DNA and Quantification of Fungal DNA Content by qPCR

Genomic DNA was extracted from medium‐sized galls at 8 dpi using the NucleoSpin Plant II Kit (Macherey‐Nagel, Düren, Germany) according to the manufacturer's instructions. Fungal and host DNA were quantified using the primer pairs for *UmPPI*, *UmRab7* and *ZmGAPDH as* described by Lanver et al. (2018).

### 4.4 Quantification of Protein, Nitrate, Ammonium and Free Amino Acid Contents

At 8 dpi, medium‐sized galls located on the middle of the blade of infected fourth leaves were excised and employed for steady‐state metabolite analysis along with samples taken from the same position and leaf number of mock‐treated control plants. Analysis of protein and nitrate was performed as described by Horst, Doehlemann, Wahl, Kahmann, et al. (2010). Extraction of leaf material and derivatization of ammonium and free amino acid with AQC (6‐aminoquinolyl‐*N*‐hydroxysuccinimidyl carbamate) was also conducted according to Horst, Doehlemann, Wahl, Kahmann, et al. (2010).

HPLC separation of AQC derivatives was performed on a Macherey‐Nagel EC 150/3 NUCLEOSHELL RP 18plus (2.7 μm, 150 mm length, 3.0 mm internal diameter) C_18_ column equipped with an EC UNIVERSAL RP guard column at a column temperature of 37°C and a flow rate of 1.1 mL/min on an Agilent 1260 Infinity II Prime UHPLC system by a ternary buffer system with buffer A (140 mM sodium acetate + 7 mM triethanolamine, pH 6.0), buffer B (99% acetonitrile) and buffer C (H_2_O_dd_). The gradient started with 98% A, 2% B, reaching 94% A and 6% B at 17 min, 88% A and 12% B at 20 min, 80% A and 20% B at 29 min, before the column was washed with 60% B and 40% C from 32 to 34 min, before the column was equilibrated to 98% A and 2% B until 38 min, which was kept for 2 min until the total run time of 40 min. Detection was performed with an Agilent G712B fluorescence detector at an excitation wavelength of 300 nm and an emission wavelength of 400 nm. For quantification, dilution series of the Pierce amino acid standard supplemented with GABA were analysed in the range of 1–200 pmol per injection.

### 4.5 Statistical Analysis

Statistical analysis of the data was performed with Graph Pad Prism (10.4.1) employing two‐way ANOVAs with Fisher's LSD post hoc tests.

## Author Contributions

**Philipp L. Lopinski:** formal analysis (lead), investigation (equal), visualisation (lead). **Christin Schulz:** funding acquisition (supporting), investigation (equal), formal analysis (supporting), resources (lead), visualisation (supporting). **Alicia Fischer:** formal analysis (supporting), investigation (supporting). **Nadine Reichl:** formal analysis (supporting), investigation (supporting). **Nadja Braun:** methodology (equal), investigation (supporting), validation (supporting). **Timo Engelsdorf:** methodology (equal), validation (supporting), writing – review and editing (equal). **Lars M. Voll:** conceptualization (lead), data curation (lead), funding acquisition (lead), formal analysis (supporting), project administration (lead), resources (supporting), supervision (lead), validation (lead), visualisation (supporting), writing – original draft preparation (lead), writing – review and editing (equal).

## Conflicts of Interest

The authors declare no conflicts of interest.

## Supporting information


**Figure S1:** mpp70148‐sup‐0001‐FigureS1.docx.

**Figure S2:** mpp70148‐sup‐0002‐FigureS2.docx.

**Figure S3:** mpp70148‐sup‐0003‐FigureS3.docx.

**Figure S4:** mpp70148‐sup‐0004‐FigureS4.docx.

**Figure S5:** mpp70148‐sup‐0005‐FigureS5.docx.

**Table S1:** mpp70148‐sup‐0006‐TableS1.docx.

**Table S2:** mpp70148‐sup‐0007‐TableS2.docx.

**Table S3:** mpp70148‐sup‐0008‐TableS3.xlsx.

**Table S4:** mpp70148‐sup‐0009‐TableS4.docx.

## Data Availability

Data available on request from the authors.
