# Supplementary material for: Ustilago maydis Nit2 Regulates Nitrate Utilisation During Biotrophy and Affects Amino Acid Metabolism of Galls Under Nitrogen Depletion
Source: Mol Plant Pathol. 2025 Sep 1;26(9):e70148. doi: 10.1111/mpp.70148 (PMC12401940; doi:10.1111/mpp.70148)
Supplement: Supplementary file 1 — Figure S1: mpp70148‐sup‐0001‐FigureS1.docx. [file MPP-26-e70148-s004.docx]

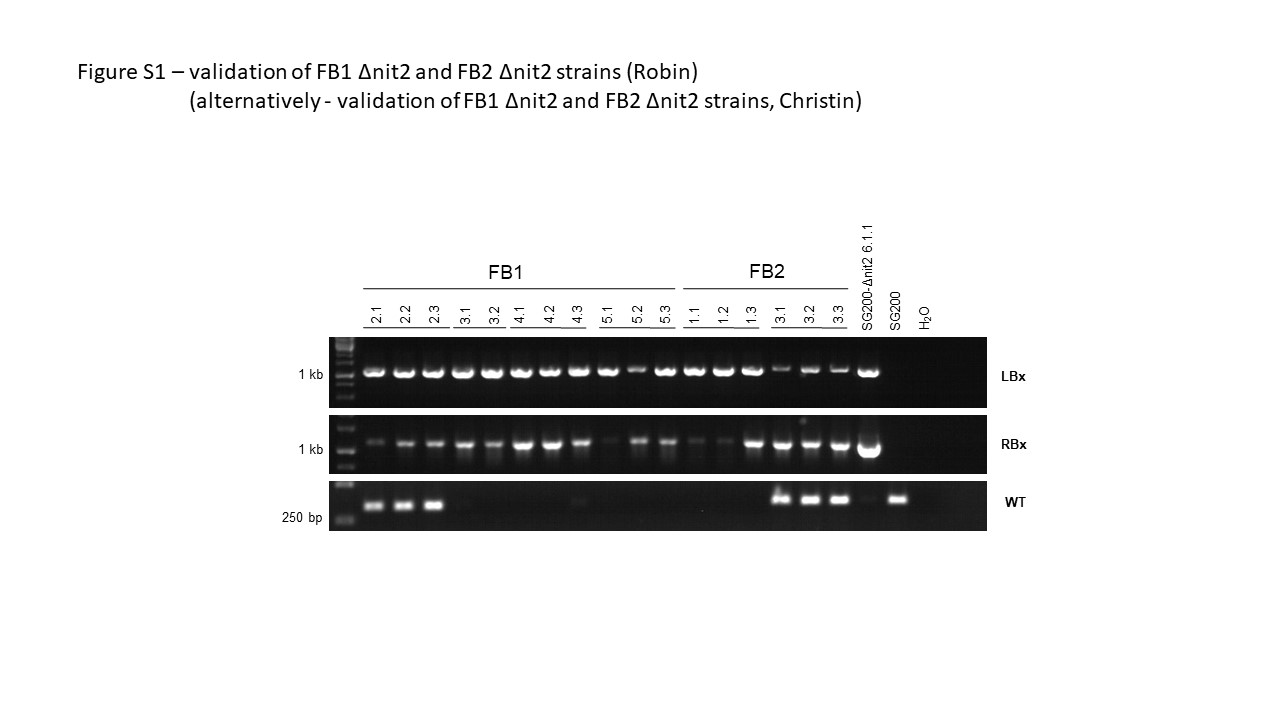


**Figure S1.** Validation of the FB1∆*nit2* and FB2∆*nit2* knockout by PCR on genomic DNA of single spore isolates.

The panels on top indicate strain (FB1 or FB2) and number of the single cell isolates tested, including one SG200∆*nit2* and SG200 control on the right end. The left flank of the k.o. cassette insertion (LBx, top panel), right flank of the k.o. cassette insertion (RBx, middle panel) and the WT allele were detected by primer pairs nit2LBx 5’-CGAGTCTTTTCAGTCTTGTCTTTC-3’ and hhn5.2 5’- CCGATGCAAAGTGCCGATAAAC-3’ (for LBx) nit2RBx 5’-GGAGTGTCACAATTTCGGCTG-3’ and hhn3.2 5’-GCTCAACTTTCATCGTGCCCAG-3’ (for RBx) and wild type Nit2WT-fw 5’- GCCTCTCGAAGAAACAGTGG-3’ and Nit2WT-rv 5’- AGAACGGGATCGACTGACAC-3’ for the WT product.
