# Supplementary material for: Ustilago maydis Nit2 Regulates Nitrate Utilisation During Biotrophy and Affects Amino Acid Metabolism of Galls Under Nitrogen Depletion
Source: Mol Plant Pathol. 2025 Sep 1;26(9):e70148. doi: 10.1111/mpp.70148 (PMC12401940; doi:10.1111/mpp.70148)
Supplement: Supplementary file 2 — Figure S2: mpp70148‐sup‐0002‐FigureS2.docx. [file MPP-26-e70148-s007.docx]

**
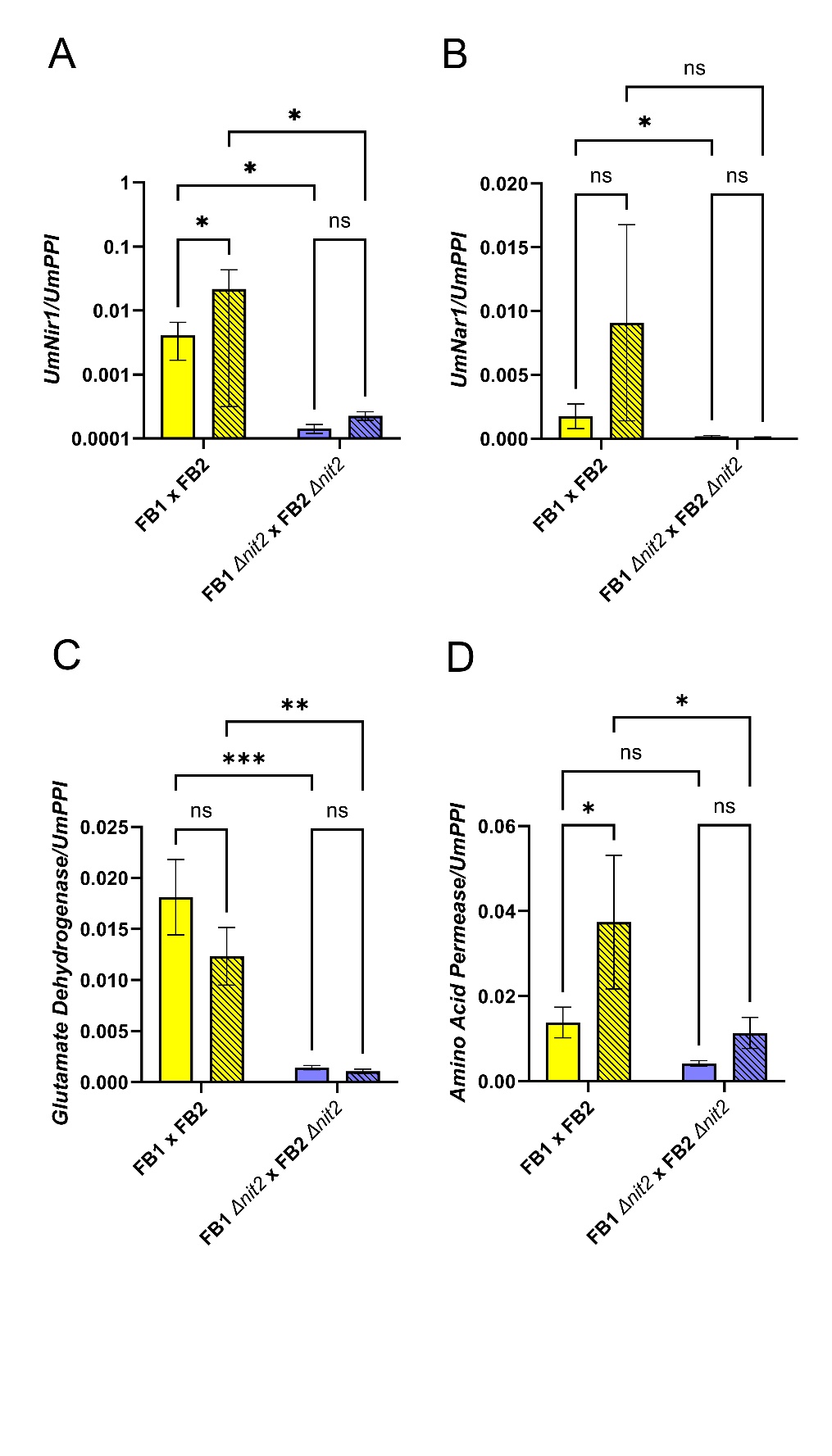
**

**Figure S2.** Nit2-dependent transcript accumulation of *U. maydis* genes in galls during biotrophy at 8 dpi.

Medium galls of comparable size were harvested at 8 dpi and transcript amounts for the *U. maydis genes* **(A)** nitrite reductase *nir1* (*UMAG_11104*), **(B)** nitrate reductase *nar1* (*UMAG_03847*), **(C)** NADP glutamate dehydrogenase (*UMAG_02801*) and **(D)** an amino acid permease (*UMAG_00056*) were quantified in a qRT-PCR relative to the *UmPPI* reference gene. Values are means of 3-5 biological replicates ± SE. Statistical analysis was conducted with a two-way ANOVA a Fisher LSD post hoc test (*P < 0.05; **P < 0.01; ***P < 0.001; ****P < 0.0001).
