# Supplementary material for: Ustilago maydis Nit2 Regulates Nitrate Utilisation During Biotrophy and Affects Amino Acid Metabolism of Galls Under Nitrogen Depletion
Source: Mol Plant Pathol. 2025 Sep 1;26(9):e70148. doi: 10.1111/mpp.70148 (PMC12401940; doi:10.1111/mpp.70148)
Supplement: Supplementary file 3 — Figure S3: mpp70148‐sup‐0003‐FigureS3.docx. [file MPP-26-e70148-s008.docx]

**
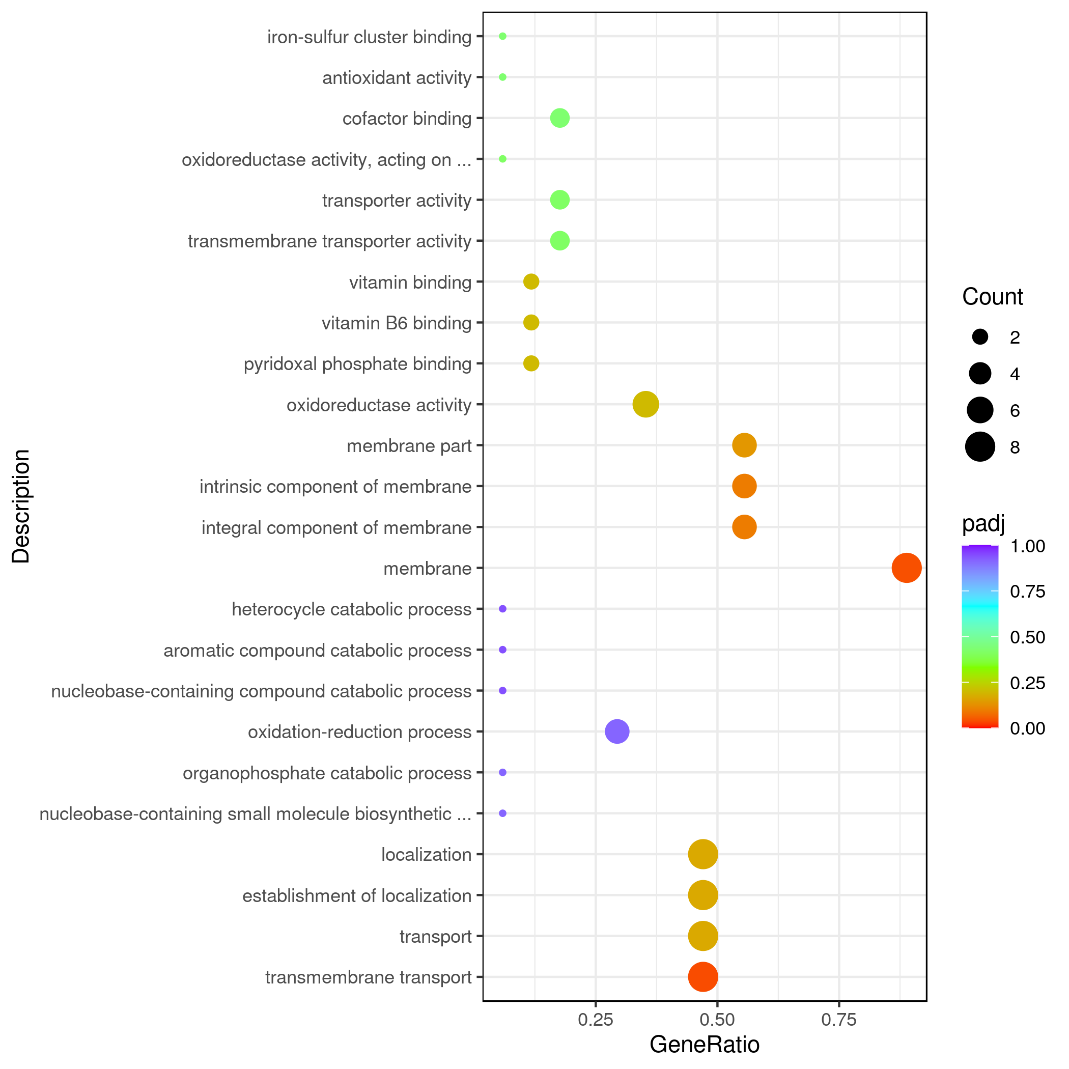
**

A

**
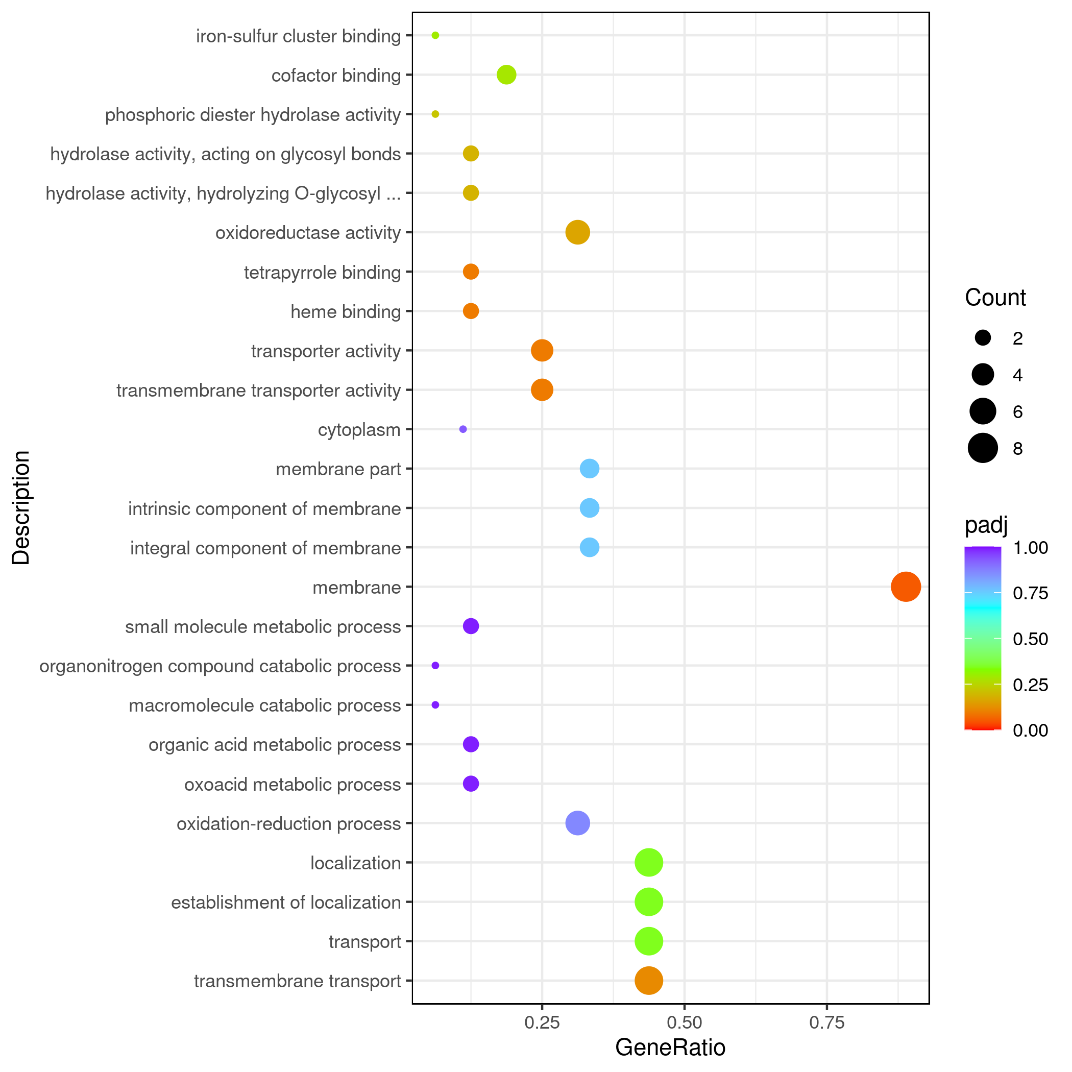
**

B

**Figure S3.** GO (gene ontology) term enrichment analysis for transcripts downregulated in ∆*nit2* compared to wild type in medium sized galls at 8dpi.

A – GO term enrichment among genes downregulated in ∆*nit2* versus wild type in 1N conditions. B – GO term enrichment among genes downregulated in ∆*nit2* versus wild type in -N conditions. Legends for gene count (bubble size) and adjusted p-value (color code) are shown next to the panels. On the x-axis, the relative fraction of downregulated relative to all genes in the respective GO term category is plotted.

Data are derived from the same experiment as the data shown in Table S3.
