# Supplementary material for: Ustilago maydis Nit2 Regulates Nitrate Utilisation During Biotrophy and Affects Amino Acid Metabolism of Galls Under Nitrogen Depletion
Source: Mol Plant Pathol. 2025 Sep 1;26(9):e70148. doi: 10.1111/mpp.70148 (PMC12401940; doi:10.1111/mpp.70148)
Supplement: Supplementary file 4 — Figure S4: mpp70148‐sup‐0004‐FigureS4.docx. [file MPP-26-e70148-s009.docx]

**
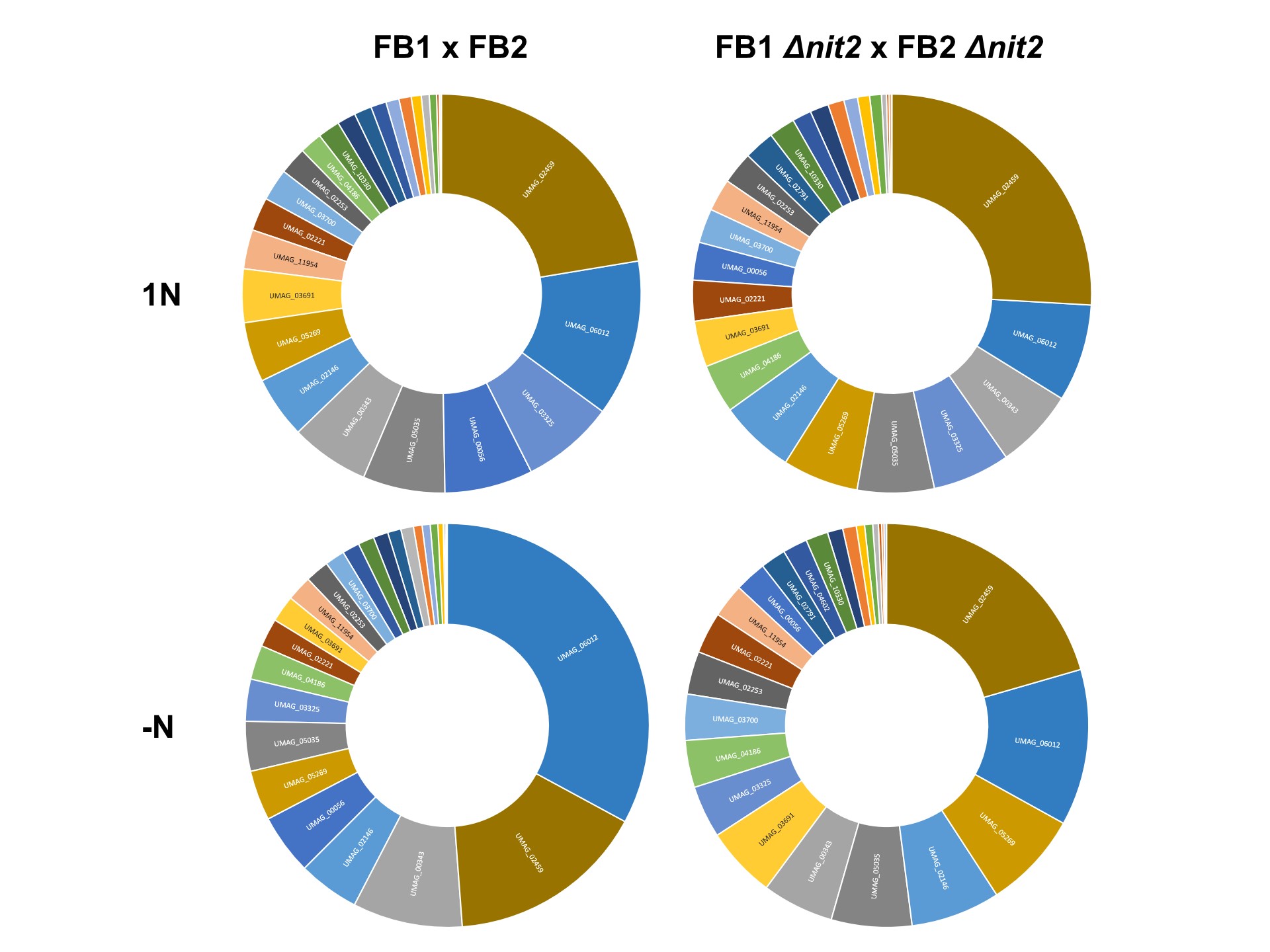
**

**Figure S4.** Relative transcript abundance of all annotated *U. maydis* amino acid transporter genes in RNA-Seq data from medium size galls at 8 dpi.

Mean FPKM values (N=2) were computed for all annotated amino acid transporter genes and the transcript amount of every individual gene relative to the total FPKM of all amino acid transporter genes is represented as pie charts. UMAG numbers are given for amino acid transporters with more than 1.5% share. 1N (upper row), -N (lower row), FB1 x FB2 (left column), FB1∆*nit2* and FB2∆*nit2* (right column). Data are derived from the same experiment as the data shown in Table S3.
