# Supplementary material for: Ustilago maydis Nit2 Regulates Nitrate Utilisation During Biotrophy and Affects Amino Acid Metabolism of Galls Under Nitrogen Depletion
Source: Mol Plant Pathol. 2025 Sep 1;26(9):e70148. doi: 10.1111/mpp.70148 (PMC12401940; doi:10.1111/mpp.70148)
Supplement: Supplementary file 5 — Figure S5: mpp70148‐sup‐0005‐FigureS5.docx. [file MPP-26-e70148-s005.docx]

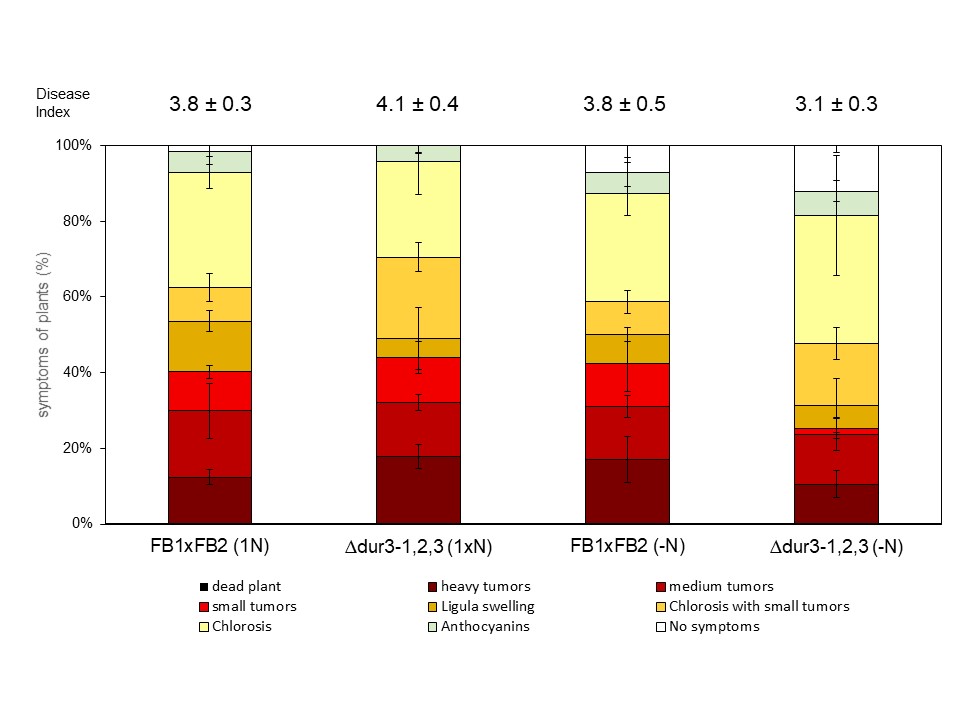


**Figure S5.** Disease index of the *dur3-1,2,3* triple knockout mutant at 8 days post infection dpi. To this end, 7 days old plants had been infected with a mixture of the indicated sporidia at a titer of OD=1 each. Mean values from four representative independent experiments with N=15-25 plants per experimental replicate and a total number of N=70-75 plants are shown ± SE. Mean values for the disease indices are given above the respective graphs ± SE.
