# Supplementary material for: Ustilago maydis Nit2 Regulates Nitrate Utilisation During Biotrophy and Affects Amino Acid Metabolism of Galls Under Nitrogen Depletion
Source: Mol Plant Pathol. 2025 Sep 1;26(9):e70148. doi: 10.1111/mpp.70148 (PMC12401940; doi:10.1111/mpp.70148)
Supplement: Supplementary file 6 — Table S1: mpp70148‐sup‐0006‐TableS1.docx. [file MPP-26-e70148-s003.docx]

**Table S1. Utilization of nitrogen sources by FB1∆*nit2* and FB2∆*nit2*** **sporidia in minimal medium.**

Growth of FB1, FB1Δ*nit2*, FB2, and FB2Δ*nit2* sporidia on minimal medium plates supplemented with selected single nitrogen sources. Sporidia dilution series of each genotype were plated and growth properties were rated 1 day after plating as normal growth (+), reduced growth (+-), strongly reduced growth (-) and no growth (--).

|  | **FB1** | **FB1*Δnit2*** | **FB2** | **FB2*Δnit2*** |
| --- | --- | --- | --- | --- |
| **ammonium** | + | + | + | + |
| **nitrate** | + | - | + | -- |
| **Ala** | + | - | + | -- |
| **Arg** | + | + | + | + |
| **Gln** | + | + | + | + |
| **Glu** | + | + | + | + |
| **Gly** | + | - | + | -- |
| **Leu** | + | - | + | -- |
| **Phe** | + | -- | + | -- |
| **Ser** | + | +- | + | - |
