# Supplementary material for: Ustilago maydis Nit2 Regulates Nitrate Utilisation During Biotrophy and Affects Amino Acid Metabolism of Galls Under Nitrogen Depletion
Source: Mol Plant Pathol. 2025 Sep 1;26(9):e70148. doi: 10.1111/mpp.70148 (PMC12401940; doi:10.1111/mpp.70148)
Supplement: Supplementary file 7 — Table S2: mpp70148‐sup‐0007‐TableS2.docx. [file MPP-26-e70148-s001.docx]

**Table S2. Verification of transcript accumulation of selected Nit2 regulated genes in sporidia by qRT-PCR.** FB1, FB2, FB1∆*nit2* and FB2∆*nit2* sporidia were transferred to nitrogen starvation (-N) or ammonium minimal medium (AMM) and harvested 2 h after transfer. Shown is the absolute fold change and standard error of –N compared to AMM control determined from two experimental replicates with three technical replicates. The normalized transcript amount of the indicates genes with *UmGAPDH* as a reference gene was determined with the primer pairs described by Horst et al. (2012).

| GeneID | Annotation | -N vs. AMM | | -N vs. AMM | |
| --- | --- | --- | --- | --- | --- |
|  |  | FB1 | FB2 | FB1∆*nit2* | FB1∆*nit2* |
| *UMAG_01756* | purine transporter | 1.96 ± 0.64 | 5.24 ± 1.71 | 0.58 ± 0.03 | 1.05 ± 0.03 |
| *UMAG_04577* | urea permease *dur3* | 19 ± 3 | 944 ± 306 | 4 ± 0.4 | 2 ± 0.1 |
| *UMAG_05889* | *ump2* | 1088 ±136 | 718 ± 125 | 64 ± 1.4 | 47 ± 1.4 |
