# Supplementary material for: Ustilago maydis Nit2 Regulates Nitrate Utilisation During Biotrophy and Affects Amino Acid Metabolism of Galls Under Nitrogen Depletion
Source: Mol Plant Pathol. 2025 Sep 1;26(9):e70148. doi: 10.1111/mpp.70148 (PMC12401940; doi:10.1111/mpp.70148)
Supplement: Supplementary file 9 — Table S4: mpp70148‐sup‐0009‐TableS4.docx. [file MPP-26-e70148-s002.docx]

**Table S4. Composition of Hoagland Nutrient Solutions** used to generate the three fertilization regimes 3N, 1N and –N (left to right). Plants were watered with the solutions 2-3 times per week, according to their demand.

**Component** **Stock Solution mL Stock Solution/1L**

**3N 1N -N**

2M KNO_3_ 202g/L 7.5 2.5 0

2M Ca(NO_3_)_2_ x 4H_2_O 236g/0.5L 7.5 2.5 0

Iron (Sprint 138 iron chelate) 15g/L 1.5 1.5 1.5

2M MgSO_4_ x 7H_2_O 493g/L 1 1 1

1M NH_4_NO_3_  80g/L 3 1 0

1M KH_2_PO_4_ (pH 6.0) 136g/L 0.5 0.5 0.5

2M CaCl_2_ 0 0 2.5

Minors: 1 1 1

H_3_BO_3_  2.86g/L

MnCl_2_ x 4H_2_O 1.81g/L

ZnSO_4_ x 7H_2_O 0.22g/L

CuSO_4_ 0.051g/L

Na_2_MoO_4_ x 2H_2_O 0.12g/L
